# Redundancy-Aware Topic Modeling for Patient Record Notes

**DOI:** 10.1371/journal.pone.0087555

**Published:** 2014-02-13

**Authors:** Raphael Cohen, Iddo Aviram, Michael Elhadad, Noémie Elhadad

**Affiliations:** 1 Department of Computer Science, Ben Gurion University, Beer Sheva, Israel; 2 Department of Biomedical Informatics, Columbia University, New York, New York, United States of America; Memorial Sloan Kettering Cancer Center, United States of America

## Abstract

The clinical notes in a given patient record contain much redundancy, in large part due to clinicians’ documentation habit of copying from previous notes in the record and pasting into a new note. Previous work has shown that this redundancy has a negative impact on the quality of text mining and topic modeling in particular. In this paper we describe a novel variant of Latent Dirichlet Allocation (LDA) topic modeling, Red-LDA, which takes into account the inherent redundancy of patient records when modeling content of clinical notes. To assess the value of Red-LDA, we experiment with three baselines and our novel redundancy-aware topic modeling method: given a large collection of patient records, (i) apply vanilla LDA to all documents in all input records; (ii) identify and remove all redundancy by chosing a single representative document for each record as input to LDA; (iii) identify and remove all redundant paragraphs in each record, leaving partial, non-redundant documents as input to LDA; and (iv) apply Red-LDA to all documents in all input records. Both quantitative evaluation carried out through log-likelihood on held-out data and topic coherence of produced topics and qualitative assessement of topics carried out by physicians show that Red-LDA produces superior models to all three baseline strategies. This research contributes to the emerging field of understanding the characteristics of the electronic health record and how to account for them in the framework of data mining. The code for the two redundancy-elimination baselines and Red-LDA is made publicly available to the community.

## Introduction

The information contained in the electronic health record for a given patient record is quite redundant. In the clinical community there is some discontent with copying and pasting [1], [2] and its negative impact on quality of clinical documentation. In previous work, we have shown through a quantitative analysis that redundancy also hurts standard text-mining tools, such as collocation identification and topic modeling [3]. In this paper, we focus on topic modeling for clinical notes.

Topic Modeling with Latent Dirichlet Allocation (LDA) [4] is a popular unsupervised method for discovering latent semantic properties of a document collection. Topic modeling has been shown to help in large number of tasks, including document classification and clustering, multi-document summarization [5], search [6], document labeling [7], [8], and information extraction [9]. LDA is known to be sensitive to noise: for example, it is standard practice to remove stop words before applying LDA, and Walker et al. showed that LDA underperforms on documents noisy with optical character recognition (OCR) errors [10]. The measure of LDAs sensitivity to different kinds of noise is not well understood, especially as various methods are used for evaluating the produced topic models [11], [12].

In the biomedical domain, probabilistic graphical models like LDA are an emerging technology only recently applied to EHR data [13]–[17], biomedical corpora [18]–[20], and consumer health resources [21]. While these initial results are promising on EHR data, initial research suggests that the specific characteristics of the clinical language affect the methods and results of these techniques [3], [22], [23].

In this paper, we describe a novel variant of LDA topic modeling, Red-LDA, which takes into account the inherent redundancy of clinical notes within a given patient record, and produces better topic models, as shown through quantitative and qualitative evaluation. We experiment with three baselines and a novel variant of LDA to handle redundancy: (i) apply vanilla LDA to all documents in all input records; (ii) identify and remove all redundancy by chosing a single representative document for each record as input to LDA; (iii) identify and remove all redundant paragraphs in each record, leaving partial, non-redundant documents as input to LDA; and (iv) apply Red-LDA to all documents in all input records.

### Problem Analysis

Characterizing the level of information redundancy between two sentences or two documents can be complex, and in fact has been a topic of study in paraphrasing, information fusion, and textual entailment. In this paper, redundancy is defined through a string similarity metric, very much the same way two sequences in bioinformatics are considered to have a high alignment score. Corpora of patient notes, which contain much copy-and-paste within the notes of a given patient, or document collections of news stories about the same events contain large amount of such textual redundancy. As such simple string similarity metrics are enough to characterize their levels of redundancy.

While in many natural language processing applications, redundancy is considered useful and its presence in a document collection is leveraged [24], in bioinformatics, for instance, it is standard practice to remove redundant sequences from databases [25] or to adjust for such sequences when using Gibbs sampling [26]. In Cohen et al. [3] experiments indicate that patient record redundancy negatively affects data-driven methods, and topic modeling in particular.

Let us try to understand where the LDA model assumptions are invalidated when applied to redundant data. The LDA generative model assumes that a document is produced by (i) sampling a Dirichlet distribution for topic mixture in the document, and then (ii) producing each word by choosing a topic from the document-level topic distribution and sampling the selected topic to produce the word. Copy-paste redundancy means that some of the words in a document are not sampled from the documents topics but instead are copied from another document. For example, consider two documents 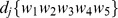 and 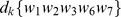, where 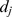 was created by the basic LDA generative process, while 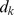 was created by copying 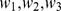 from 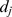 and then sampling 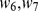 using the basic generative process. We would expect the copied words to retain their prior topic assignment (that is, the occurrences of 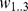 in 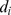 and 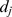 should share the same topic assignment). In practice, however, the LDA sampling algorithm may assign them to different topics. Even when the copied words are assigned to the same topic, the weight of copied words in their topic is abusively increased. If a word of low probability in topic 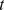 is sampled from 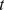 once and duplicated through copy-paste into many documents, our estimate of topic 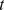 would be misled to give that word higher probability within 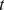. Such an approach would lead to over-fitting the model to observed data, assuming the probability to copy segments is not directly dependent on their topical relevance.

The main contribution of this paper is a variant of LDA, Red-LDA, which takes into account the fact that words are copied from a source document in the patient records. The next section shows the results of our experiments comparing Red-LDA to three topic-modeling baselines.

## Results

To assess the value of handling redundancy explicitly as part of the topic modeling task of clinical notes, we conducted a comparison of the redundancy-aware LDA (Red-LDA) to alternative methods according to two quantitative established metrics for evaluation of topic modeling – log-likelihood and topic coherence – and a qualitative review of generated topics by clinical experts.

### Quantitative Analysis

Topic models were learned using four methods on the same training set: vanilla LDA, DeleteDoc-LDA, DeleteWord-LDA, and Red-LDA.

#### Log-likelihood

**Figure 1:**
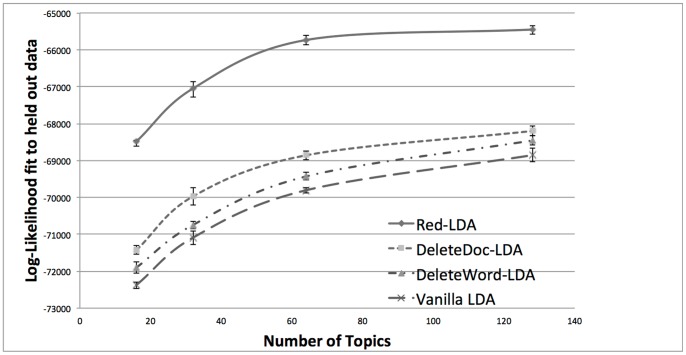
Comparison of four models according to log-likelihood of held-out data. Red-LDA performs the best, well outside the deviations of the three other models. Training vanilla LDA on just 40% of the documents (DeleteDoc-LDA) produced a better model than training on the entire redundant corpus (Vanilla LDA).

The models produced on the training set were used to produce topic assignments for the held-out documents, and log-likelihood fit of the different models were calculated. All the experiments were repeated three times and the average likelihood is reported (we report standard deviation over 3 runs). Figure 1 shows the results. The worse performance is obtained for Vanilla LDA trained on the original, redundant corpus. DeleteWord-LDA, which discarded redundant words in the input documents, produces slightly better models. In the DeleteDoc-LDA version, the resulting, non-redundant corpus contained only 40% of all documents in the original corpus. Yet, the topic it generates are superior to the ones generated by the vanilla LDA trained on the redundant, original corpus. DeleteDoc-LDA also outperforms DeleteWord-LDA, which confirms the hypothesis that discarding parts of documents rather than whole documents might hurt overall coherence of a corpus. Finally, Red-LDA outperforms all baseline methods.

#### Topic coherence

**Figure 2:**
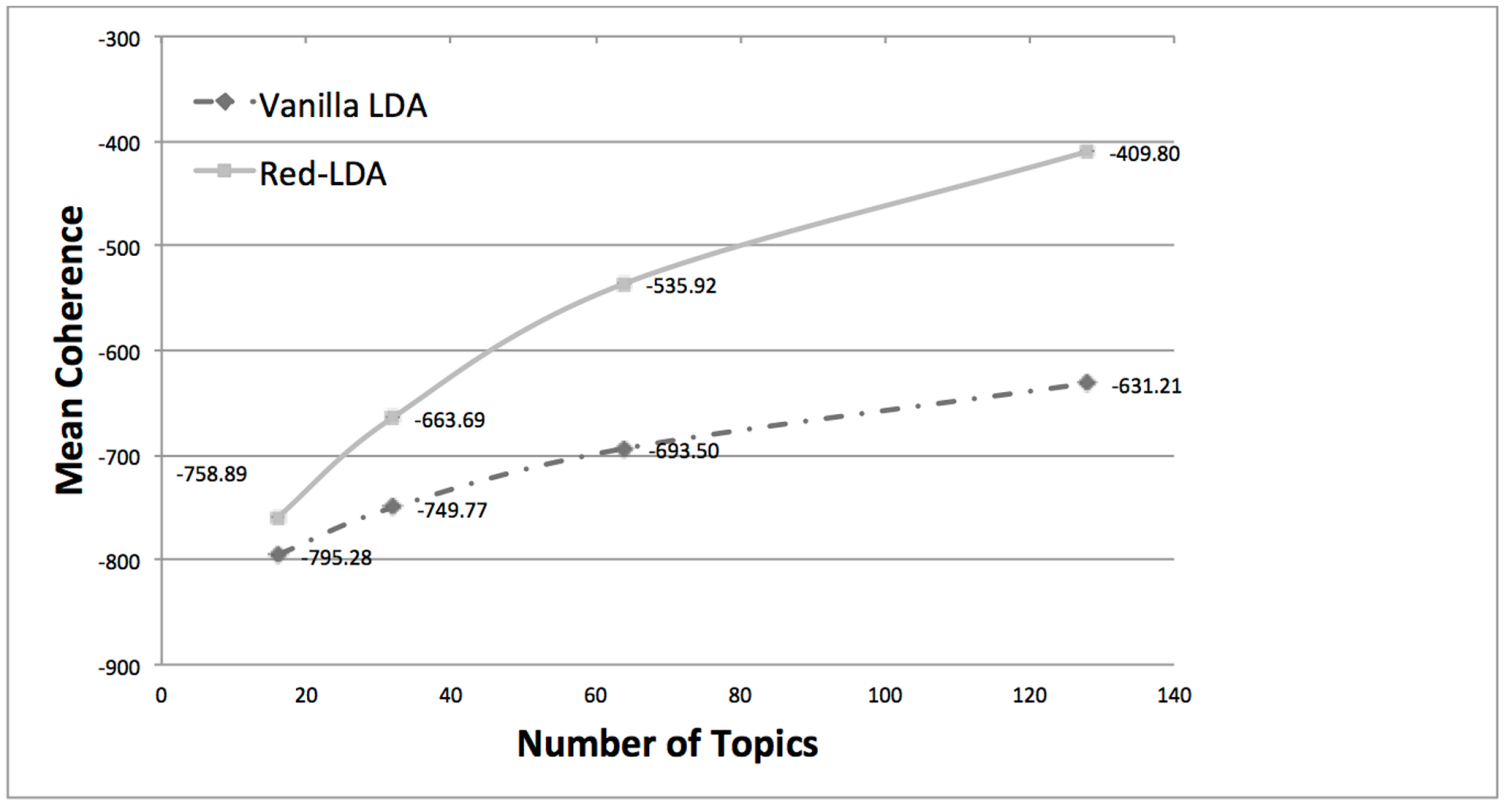
Comparison of Vanilla LDA and Red-LDA according to topic coherence. Red-LDA produces more coherent topics than the baseline.

We use the topic coherence score for evaluating the quality of topics produced by the Red-LDA and Vanilla LDA methods [12], state-of-the-art method for evaluation (see [11] for earlier work). This measure is based on the co-document frequency of the pairs of the most words probable words in each topic. Thus, the higher the topic coherence metric for a set of topics, the higher quality the topics from a semantic standpoint. All the experiments were repeated three times and the average coherence is reported. Figure 2 indicates that Red-LDA produces topics that are more coherent than the Vanilla LDA ones.

### Qualitative Analysis

**Figure 3:**
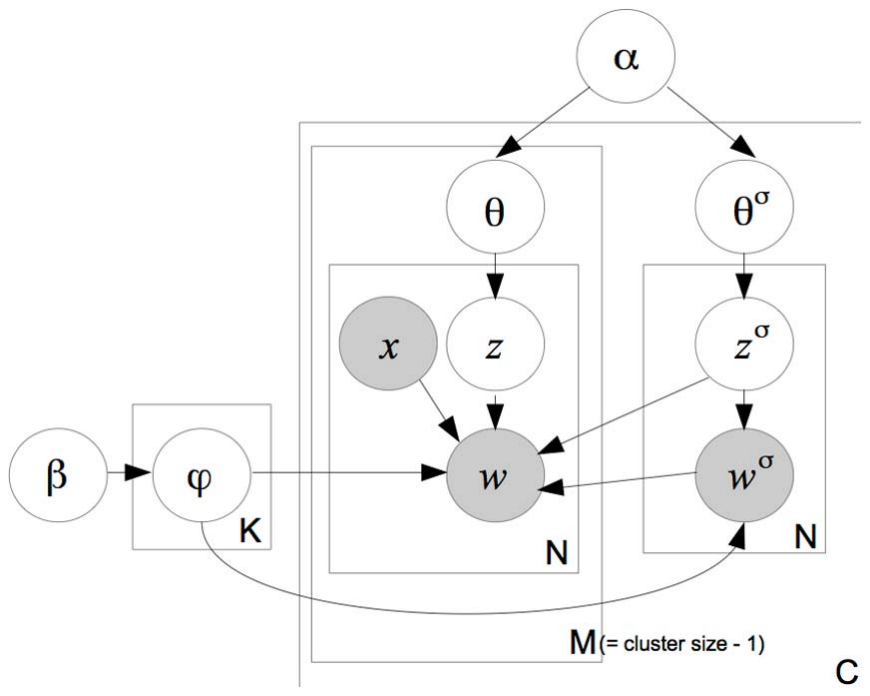
Topics learnxled by Red-LDA (top) and Vanilla LDA (bottom) on the EHR corpus. Both topics are about breast cancer (ca is an abbreviation for cancer). The Vanilla LDA topic, however, contains unrelated yet highly ranked words (*e.g.*, eye, glaucoma, colonoscopy, albuterol). The Red-LDA topic was preferred by the domain experts.

To compare the quality of topics across models, we experimented with models learned with 50 topics, both for vanilla LDA and Red-LDA. Out of the 50 topics for each model, we fist aligned the topics learned using Jensen-Shannon divergence. The JS divergence identified 25 topic pairs with high enough similarity. First, the 25 topic pairs were presented to domain experts for review. For instance, the two clouds in Figure 3, representative of a topic produced by Red-LDA and one produced by vanilla LDA, were shown side by side to the domain experts (along with the ranked list of words for each word cloud/topic). Other learned topics and their comparison to vanilla LDA are available at http://www.cs.bgu.ac.il/~cohenrap/RedLDA. The experts were asked to chose which topic they felt was better quality, where a quality topic is defined both as representative of a clinical concept and as pure (*i.e.*, with little words that did not belong to the concept represented by the topic). Second, the domain experts were presented with the remaining 50 individual topics (25 from vanilla LDA and 25 from Red-LDA), which were not aligned to any other topic according to their JS divergence. The experts were asked to rate on a Likert scale each topic’s quality according to the same quality criteria as for the pair comparison. The goal of the side-by-side comparison was to assess for the same topics, which model produced higher quality. The goal of the individual topic rating was to determine whether any model was able to identify high-quality topics, which the other model had missed (since they were not aligned to any other topic).

When two domain experts compared the 25 aligned topic pairs, the Red-LDA topic was preferred in 13 cases, while the vanilla LDA topic was preferred in 4 cases. Furthermore, in the case of these four cases, the two experts disagreed in their assessment and expressed low confidence with their choice. When comparing the 25 non-aligned topics based on their likert score, on average Red-LDA topics were rated as higher quality than vanilla LDA topics 3.1 *v.s.* 2.7 (p = 0.1 on this relatively small sample). Overall, Red-LDA modeling tends to produce higher quality topics when interpreted in a clinical context.

## Discussion

The experiments on different topic modeling techniques and their handling of the inherent redundancy of clinical notes indicate that while redundancy is harmful to traditional topic modeling technique, it is possible to mitigate its effect. Of all methods, Red-LDA yields the best topic models, both from a quantitative and a qualitative standpoint. Interestingly, the best-performing method does not discard any information, like the two baselines which outperform the vanilla LDA.

When examining the underlying data, we observe, for instance, that a very small number of patients in our data set had a particularly high number of notes (and thus high level of redundancy) and had both breast cancer and glaucoma (two unrelated clinical conditions). The vanilla LDA modeling was overwhelmed with the number of co-occurrences of the two conditions in the corpus overall because each note was considered equally, independently of whether they belonged to the same cluster of patient notes.

The three methods, DeleteDoc-LDA, DeleteWord-LDA, and Red-LDA, all rely on a preprocessing step to identify the “source” document in each record. There are many possible methods for choosing such a document (document size, metadata such as time stamps or average distance to the other documents within the cluster) or even to generate a projected source document to best represent the shared information between documents in the cluster. These methods and effects on different corpora may be studied in depth in the future.

As more and more corpora are made available to researchers, one can characterize them through different statistical properties (*e.g.*, size of vocabulary, token-type ratio). One property which has received little explicit attention is the amount of redundancy inherent to a corpus. This research contributes primarily to the emerging field of understanding the characteristics of patient records, their impact on mining techniques, and how to correct for them [27].

Our findings on collection-level redundancy and text mining, are also pertinent to other domains. Redundancy, similar to the copy-and-paste redundancy of patient record, has been observed in Twitter [24] (re-tweeting messages with only small changes), repeated spam messages which vary only slightly, and template texts, such as movie and theater listings [12]. Redundancy occurs also in collections with longer documents. In the news domain, for instance, the same story authored by a news agency is reported by a number of sources, resulting in clusters of documents likely to be rich in redundancy [28]. Redundancy increases as events progress through time and stories get incrementally updated, very much like a patient record gets incrementally updated through time. As more and more social media emerges online, text mining methods must pay specific attention to the statistical parameters of text collection, and empirically analyze the implications of skewed distributions on data exploration methods. Our experiments indicate that (i) when string-level redundancy is prevalent, it has critical impact on the quality of topic modeling; and (ii) when handled within the topic learning, better quality topics can be produced than by ignoring the redundancy or discarding it harshly through pre-processing. This suggests, that not all redundancy is bad in text mining, and confirms that some redundancy indicate topic centrality and is thus important to keep during text mining activities, while some redundancy is just an artifact of documentation. We are interested in further understanding the relation between repetition and topical centrality across different domains in further experiments.

## Materials and Methods

This section describes the data set on which we conducted our experiments, along with two baseline methods, that handle redundancy naively (Redundancy Elimination and DeleteLDA), and the algorithm for Red-LDA.

### Corpus of Patient Records

The dataset for our experiments is a large collection of patient records from the Columbia University Medical Center. IRB approval was obtained from the Columbia University Medical Center Institutional Review Board. As part of the approved IRB protocol, an application for waiver of authorization was filled and approved, as we investigate algorithms for learning statistical models across a very large population and historical data across many years, and thus obtaining individual consent would be impracticable. We collected the notes for 1,247 distinct patients. These 1,247 are all the patients who visit the outpatient clinic at the medical center and who also visit a nephrologist for kidney problems, and have overall at least three visits in their longitudinal record. As such, the data set represents a somewhat homogeneous set of patients from a clinical standpoint. In our experiments, we divided the corpus into a training set and held-out test set of 250 non-redundant documents from 250 patients not included in the training set. The assignment of the patients into the training or testing set was carried out randomly.

In the first visit to a physician, a patient note is authored in a “natural” generative process. Later notes, however, contain large sections of copy-paste material. The corpus we analyzed contains 8,557 notes, corresponding to 6M tokens. It contains notes from 1,247 distinct patients; therefore, most notes for the same patients are likely to share some redundancy with another note in the corpus. It is important to note, however, that there is a large amount of variation in the number of notes per patients (in other words, some patients are over-represented in the corpus compared to others).

To characterize the amount of redundancy present in the EHR corpus, we sampled same-patient notes and computed the Smith-Waterman alignment of the entire document. The average similarity between same-patient notes was 29%. 25% of the notes shared similarity of over 50% to another note in the corpus, confirming the notion that the EHR corpus is a naturally occuring, highly redundant corpus.

### Input to Topic Modeling and Preprocessing

For all methods to learn a topic model, the input is a collection of patient records. A patient record is defined as a set of documents, or notes, written by the clinicians.

Given an input patient record in the collection of records, we use the online fingerprinting method described in [3] to quantify the amount of redundancy in a record through aligning all strings in all documents in the record. The fingerprinting can thus identify in a record which strings are unique within the record and which are redundant throughout the record.

### Two Baselines Through Redundancy Elimination

We present first two naïve approaches for solving the problem of redundancy in a given collection of records. Both methods rely on a fingerprint preprocessing. The first method, which we call DeleteDoc-LDA, simply removes the redundant documents from the corpus: for each patient record, a source document is chosen to represent the record as a whole, and the rest of the record is discarded. The second method, which we call DeleteWord-LDA, chooses a source to represent the cluster and deletes the redundant word from the other documents and adds these redundancy free documents to the corpus (*i.e.*, only redundant portions of documents are discarded instead of the entire document like in the first method). In both methods, vanilla LDA is then applied to the resulting corpora to learn topics.

#### DeleteDoc-LDA

Given a training set of patient records, DeleteDoc-LDA consists of the following steps: (i) for each patient record in the training set, identify a source document that is representative of the record according to the fingerprinting preprocessing; (ii) discard all other documents in the record; and (iii) apply vanilla LDA to the source documents only.

The method of discarding all redundant documents is a drastic way to ensure that there is no redundancy in the input corpus to LDA. Since most of the redundant words in the corpus are removed, this solves the two problems we identified in the previous section of topic mismatch between identical tokens (produced from the same topic and copied) and skewed estimation of the weights of replicated words in different topics.

However, this approach has serious drawbacks if the resulting corpus is not large enough. For instance, if documents 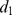 and 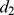 share a copy-pasted section of 30% of the two documents, removing one of the documents means losing the 70% non-redundant data in it. In addition, this method simply ignores the fact that segment repetition may be an indication of its topical relevance. The relation between repetition and importance has two aspects: when a segment is repeated, some of this repetition may be warranted by key terms, and reflect their importance. Other terms, however, within the repeated segment may simply be attached syntactically to the key terms, and their repetition is not indicative of their relevance. The second aspect is that, in certain domains like the clinical domain, repetition is not always an indication of topical centrality: the presence of copy-paste redundancy is an artifact of documentation practices and the software that clinicians use to author notes.

#### DeleteWord-LDA

This method mitigates the redundancy by aligning the documents in the record containing the redundancy to the representative source document in the record, and then removing the copied words from the other documents (instead of whole documents, like in DeleteDoc-LDA). This solves the topic mismatch problem and the skewed estimation of weights since the redundant tokens are removed. However, it leaves the documents other than the resprentative missing some of the context of the document. If documents 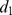 and 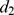 share a copy-pasted section of 30% of the documents, and 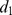 is the represantative, we will delete 30% of 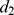 which were copy-pasted. This may harm the topic estimation in 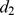, as the topic weigths in the new document do not include the removed sections.

### Redundancy-Aware LDA: Red-LDA

The approach for Red-LDA differs from the two previously described methods, as there is no data in the input records is discarded prior to learning topics. Instead, the redundancy is handled as part of the model.

**Figure 4:**
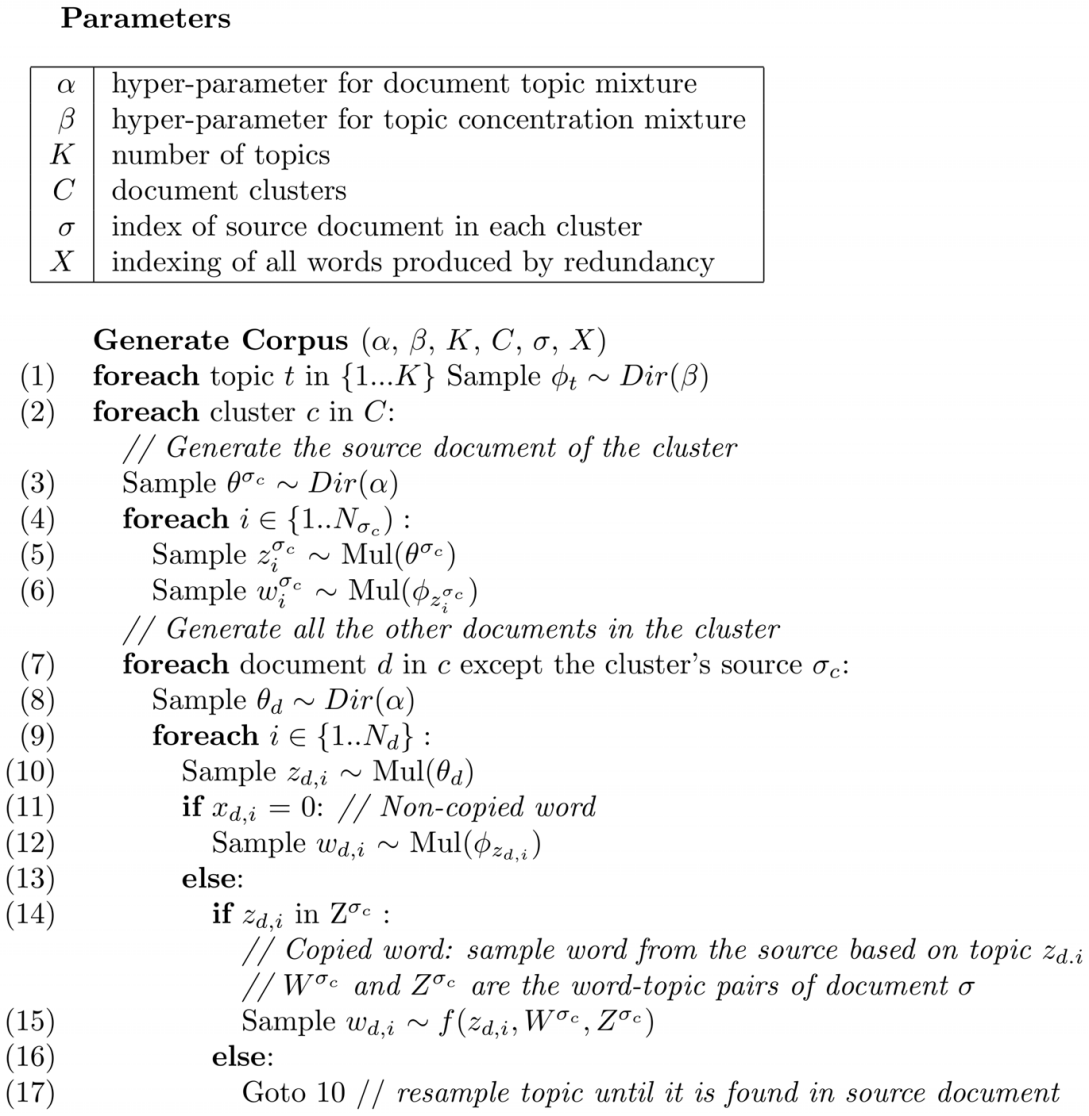
Red-LDA graphical model. Patient records are collections of documents (plate C): of a source document and the rest of the record (M documents).

Red-LDA is a probabilistic graphical model for generating a collection of records, which share different levels of redundancy. Like in LDA, each document is produced by a mixture of topics and each token is produced by one topic. Unlike LDA, Red-LDA assumes that each document belongs to a predefined cluster (*i.e.*, a patient record) and some of its tokens are sampled from another document, the source document for the record, as identified by fingerprinting preprocessing step. The graphical model for Red-LDA is shown in Figure 4.

In each cluster of documents (or record) 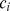, one document 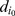 is the original “source” document and the others 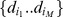 are documents containing redundant parts copied from the source 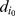. Document 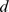 is defined as a collection of tokens 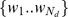, where 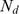 is the document length and each 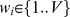 where 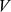 is the vocabulary size. Topic 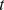 is defined as 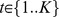 where 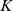 is the number of topics.

#### Generative process

**Figure 5:**
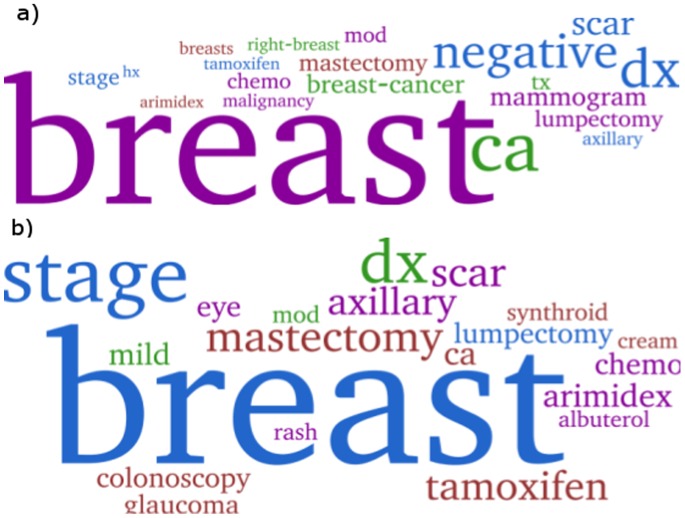
Red-LDA generative story.

The schematic description of the generative story is given in Figure 5. To generate the corpus, we iterate over the patient records in the training set, each with a set of input documents. As such, the number of clusters is predefined, as is the number of documents in each cluster. For each cluster, we select one document as a source document. In our current implementation, the source document is selected as the longest note in the patient record. For the source document, its words are generated as per the standard LDA process – draw a multinomial distribution of topics for the document from a Dirichlet distribution; then for each word in the document, sample a topic from the document topic distribution, then sample a word from the topic. The change from LDA lies in the other documents in the cluster: within these documents, we first observe whether a word is copied from the source document in the cluster (this information comes from the fingerprinting pre-processing step). If the word is not copied, we generate it as usual by sampling a topic then the word from the topic. Else, we sample a topic from the document topic distribution, and then sample the source document for a word that has been assigned the same topic in the source document of the cluster.

By construction, the words that are copied from a source document (as determined by the fingerprinting step) are assigned the same topic in their copied occurrences as in the source document. Note also that because copied words must be assigned a topic that occurs in the source document, the distribution of topics in the copied documents is influenced by the distribution of topics in the source document (lines (14)-(17) in the generative story).

#### The algorithm

Given a collection of patient records, each record consisting of a group of documents where one can be considered the source for each patient and the other the rest of the record. Given such a collection, the algorithm for Red-LDA is as follows. The graphical model is shown in Figure 4. Sampling of a non-redundant word is the same as in the baseline Gibbs-Sampling for LDA:
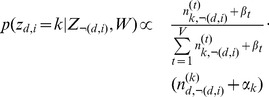
(1)


When a redundant word is encountered, the topic is sampled from the topic distribution of identical tokens in the source document:

**Case** 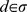 or 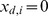:
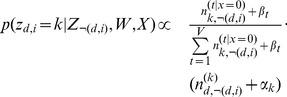
(2)Where 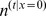 is like 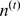 with the difference that words whose 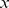 has the value 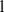 (*i.e.*, are copied) are not counted.

**Case** 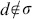 and 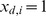:
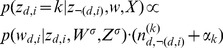
(3)Where 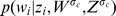 is the probability of the word 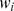, which is known as having the topic 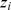, and being copied from the source document 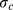 of the cluster 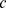 where the word-topic pairs 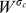, 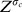 have already been sampled. In our model we have:
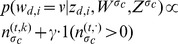
(4)Where 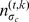 is the number of occurrences of the word 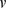 assigned to topic 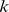 in the source document 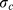 of the cluster. The variable 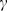 is a smoothing parameter with value between 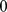 and 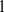.

The topic assignment for redundant words is not counted for the approximation of the topic-word multinomial distribution (in order to avoid giving a redundant word excess weight in the topic distribution, as described in the Problem Analysis Section).

#### Running time

Red-LDA differs from vanilla LDA in two ways: (i) it requires pre-processing to identify source documents; and (ii) copied tokens are sampled from the source. The running time of the pre-processing step is very low [3] (less than a minute on a standard PC for our dataset). The change during sampling ends up speeding up the LDA algorithm, as for redundant tokens we only sample their topic proportion in the source instead of using the topic-word and topic-document counts for all topics. Using 2,000 iterations for both vanilla LDA and Red-LDA on a standard PC for our dataset, vanilla LDA took 8.3 minutes, while Red-LDA took 7.35 minutes.

### Experimental Setup

The code for the fingerprinting preprocessing step is made publicly available at https://sourceforge.net/projects/corpusredundanc. We used the Gibbs Sampling code by Heinrich [29] for the vanilla LDA. Red-LDA was implemented over the Heinrich code and is available at https://sourceforge.net/projects/redlda. The fingerprinting preprocessing was applied to the training set of records, with a maximum redundancy threshold set to 15% for all methods, DeleteDoc-LDA, DeleteWord-LDA, and Red-LDA.
