# Correction: Redundancy-Aware Topic Modeling for Patient Record Notes

**DOI:** 10.1371/journal.pone.0114677

**Published:** 2014-11-24

**Authors:** The PLOS ONE Staff

The images for Figure 3 and Figure 5 were incorrectly switched. Please view the correct images and legends here.

**Figure 3:**
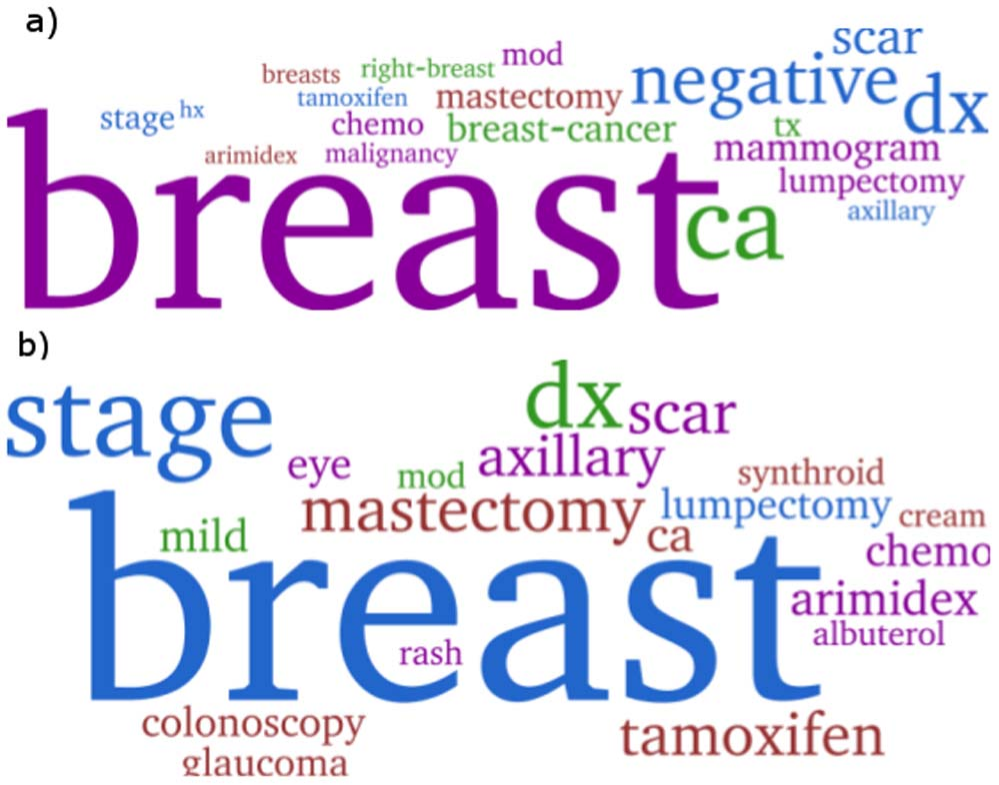
Topics learnxled by Red-LDA (top) and Vanilla LDA (bottom) on the EHR corpus. Both topics are about breast cancer (ca is an abbreviation for cancer). The Vanilla LDA topic, however, contains unrelated yet highly ranked words (*e.g.*, eye, glaucoma, colonoscopy, albuterol). The Red-LDA topic was preferred by the domain experts.

**Figure 5:**
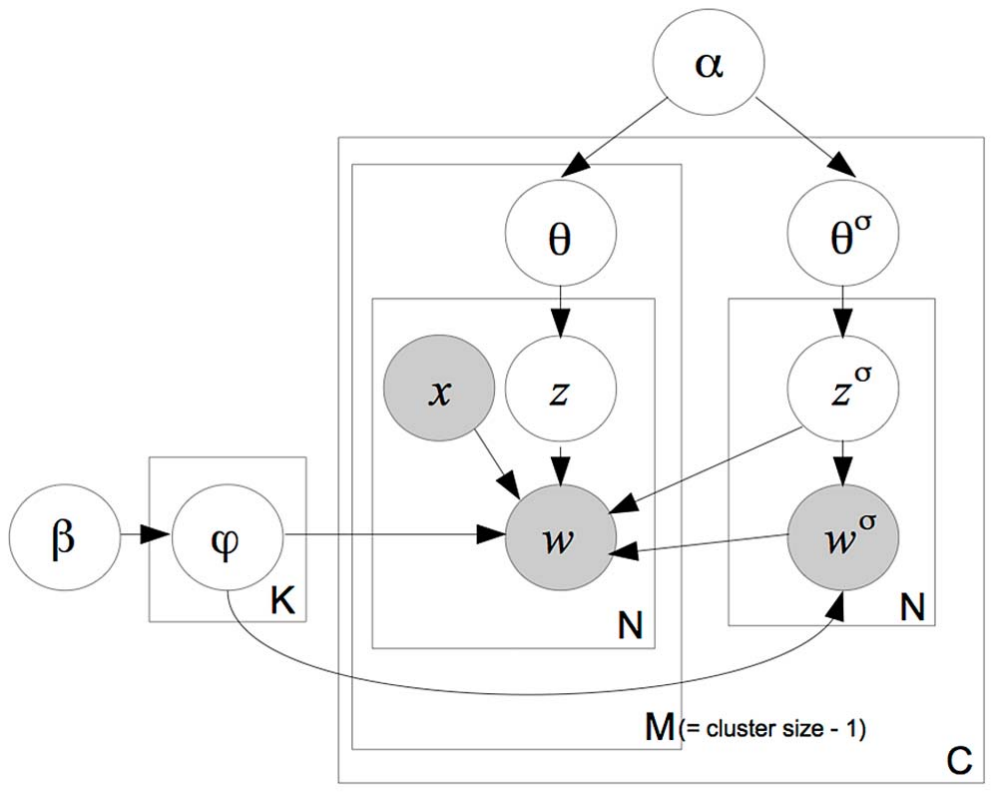
Red-LDA generative story.
